# Preliminary Evidence for an Association between LRP-1 Genotype and Body Mass Index in Humans

**DOI:** 10.1371/journal.pone.0030732

**Published:** 2012-02-08

**Authors:** Alexis C. Frazier-Wood, Edmond K. Kabagambe, Ingrid B. Borecki, Hemant K. Tiwari, Jose M. Ordovas, Donna K. Arnett

**Affiliations:** 1 Department of Epidemiology, School of Public Health, University of Alabama, Birmingham, Alabama, United States of America; 2 Section on Statistical Genetics, School of Public Health, University of Alabama, Birmingham, Alabama, United States of America; 3 Office of Energetics, School of Public Health, University of Alabama, Birmingham, Alabama, United States of America; 4 School of Public Health, Nutrition Obesity Research Center, University of Alabama, Birmingham, Alabama, United States of America; 5 Division of Statistical Genomics, Department of Genetics, School of Medicine, Washington University, St. Louis, Missouri, United States of America; 6 Nutrition and Genomics Laboratory, Jean Mayer United States Department of Agriculture Human Nutrition Research Center on Aging, Tufts University, Boston, Massachusetts, United States of America; 7 The Department of Epidemiology and Population Genetics, Centro Nacional Investigación Cardiovasculares (CNIC) Madrid, Spain; 8 IMDEA Food, Madrid, Spain; City of Hope National Medical Center and Beckman Research Institute, United States of America

## Abstract

**Background/Aims:**

The LDL receptor-related protein-1 gene (*LRP-1*) has been associated with obesity in animal models, but no such association has yet been reported in humans. As data suggest this increase in fat mass may be mediated through a mechanism involving the clearance of plasma triglyceride-rich lipoproteins (TGRL), where the LRP interacts with apolipoprotein E (ApoE) on chylomicron remnants, we aimed to examine (1) whether there was an association between 3 single nucleotide polymorphisms (SNPs) on *LRP-1* with body mass index (BMI) and (2) whether any association between *LRP-1* SNPs and BMI could be modified by polymorphisms on the *ApoE* gene when comparing the wild type ε3/ε3 genotype against mutant *ApoE* allele (ε2/ε4) carriers.

**Methods/Results:**

We used data from 1,036 men and women (mean age±SD = 49±16 y) participating in the Genetics of Lipid Lowering Drugs and Diet Network (GOLDN) Study. Mixed linear models, which controlled for age, sex, alcohol intake and smoking, as well as family pedigree and center of data collection were calculated. Models that used *LRP-1* genotype as a predictor of BMI revealed that individuals who were homozygous for the minor allele at the LRP-1 I10701 locus had BMIs, on average, 1.03 kg/m^2^ higher than major allele carriers (*P* = 0.03). In subsequent mixed linear models that included main effects of *LRP-1* I10701 SNP and *ApoE* alleles, and an interaction term the two genotypes, there was no interaction detected between the *LRP-1* I70701 genotype with either the ApoE ε2 or ε4 allele carriers (*P*>0.05).

**Conclusions:**

This has implications for starting to understand pathways from genotype to human BMI, which may operate through TGRL uptake at the LRP-1 receptor. This may pave the way for future research into individualized dietary interventions.

## Introduction

The role of the LRP, alongside that of the LDL receptor, in the uptake of chylomicron remnants has long been established [1]. The *LRP-1* gene is expressed in a number of tissues, including the liver, the primary site of LRP-mediated chylomicron remnant clearance [2]. *In vitro LRP-1* elimination has been shown to lead to lipid depleted cells [3] and *LRP-1* knockout mice show fat storage differences compared to wildtype mice, although results have been bidirectional [3]–[6]. Changes in fat mass in *LRP-1* knock-out mice are thought to occur, in part, through the reduced catabolism and uptake of TGRL [4]. The reduced clearance, and subsequent accumulation of TGRL has been similarly associated with increased fat mass in humans [7], yet to our knowledge, an association between human BMI and *LRP-1* variants has yet to be reported.

To enable chylomicron remnant binding and subsequent uptake, the LRP-1 binds with ApoE on the surface of remnant particle [2], [8], [9]. Three major isoforms of ApoE exist (E2, E3 and E4) which are the products of three alleles (ε2, ε3 and ε4, respectively) at the single gene *ApoE* locus. Variations in ApoE isoforms have been associated with variation in the efficacy of ApoE binding to receptor sites [10] and, from this, with differing lipid profiles. Meta-analysis across 45 population samples confirmed the differential effects of the *ApoE* alleles on lipids; carriers of ε2 had lower, and carriers of ε4 had higher, fasting plasma cholesterol values compared to ε3/ε3 genotypes [11]. The effect of different ApoE isoforms on lipids and LRP-1 mediated TGRL uptake, indicates that *ApoE* remains a promising candidate to study for effects on BMI, in conjuction with *LRP-1* polymorphisms.

The first aim of these analyses was to evaluate an association between three polymorphisms on the *LRP-1* gene and body mass index (BMI) in humans, using a population sample of 1,036 men and women between the ages of 18–87 years. The second aim was to examine whether any association between BMI and *LRP-1* identified in our sample was modified by *ApoE* allelic variants.

## Materials and Methods

### Participants

The GOLDN study population consisted of 1,328 men and women in the Genetics of Lipid-Lowering Drugs and Diet Network (GOLDN) study. All participants were white men and women recruited from Minneapolis, Minnesota and Salt Lake City, Utah. The primary aim of the GOLDN study was to characterize the role of genetic and dietary factors on an individual's response to fenofibrate. The details of the GOLDN study have been published elsewhere [12]. GOLDN consisted of an initial screening visit (visit 0) during which participants were asked to discontinue the use of lipid lowering drugs. Approximately 4 to 8 weeks later, baseline blood chemistries were measured (visit 1). A day later (visit 2) participants' blood samples were collected before (fasting) and after (postprandial) participating in a high fat meal challenge. On subsequent visits 3 and 4, fasting and postprandial blood samples were collected after a 3-week open label fenofibrate trial. For this analysis, we used blood draw and BMI data collected at visit 2. This includes data only from subjects who were willing to participate in the high fat meal intervention. The final sample consisted of 1036 individuals across 187 families; 497 men and 539 women (mean ± SD: 48.8±16.2 y of age). The protocol was approved by the Institutional Review Boards at the University of Minnesota, University of Utah, Tufts University/New England Medical Center, and the University of Alabama at Birmingham. Written informed consent was obtained from all participants.

### Data collection

Clinical characteristics including anthropometric and blood-pressure measurements were taken at the study clinics where a fasting blood sample (used for the genotyping) was also drawn, as described previously [12]. Questionnaires were administered to collect demographic data and information on lifestyle attributes and medical history.

### Measures

#### Anthropomorphic and demographic measures

BMI data were collected by trained research staff who were instructed to take weight measurements over light clothing. BMI was measured as weight in kilograms (kg) divided by height in meters squared (m^2^). Age and sex were recorded by questionnaire. Habitual alcohol (g/day) and smoking behavior (current/non) was measured by the dietary history questionnaire (DHQ).

#### Genotype data

Genomic DNA was isolated from blood samples using routine DNA isolation sets (Qiagen, Valencia, CA, USA), using a TaqMan assay with allele-specific probes on the ABI Prism 7900HT Sequence Detection System (Applied Biosystems, Foster City, CA, USA) according to the standardized laboratory protocols.

#### SNP selection

Three nonsynonymous SNPs on the *LRP-1* gene on Chromosome 12 (positions 12q13.1-q13.3) were chosen from literature reports of genetic associations or lipid-related biological function [13]–[15].These were C766T (rs1799986) in exon 3, I68477 (rs1800191) in intron 55 and I10701 (rs715948) in intron 2. SNAP from the Broad Institute (http://www.broadinstitute.org/mpg/snap/index.php) indicated that none of the SNPs were in linkage disequilibrium with r>.8.

The *ApoE* alleles were typed at the previously identified *ApoE* locus. Given that ε2 carriers and ε4 carriers have both shown effects on lipid measures in comparison to ε3, but their effects may not be in the same direction [11], we compared ε2 carriers (without including ε4 carriers) to those with the ε3/ε3 genotype and separately: ε4 carriers (without including ε2 carriers) to those with the ε3/ε3 genotype.

As we were interested at the theoretical level of how *ApoE* isoforms mediate the effects of *LRP-1* SNPs, only those *LRP-1* SNPs which showed an effect on BMI as a significant association with BMI were examined for mediation by *ApoE* alleles.

### Statistical analysis

All analyses were conducted in SAS (v9.2; SAS Institute, Cary, NC). Hardy-Weinberg equilibrium (HWE) was determined using *χ* ^2^ goodness-of-fit analysis. All SNPs were in HWE (p>.05). For genotype-phenotype associations examining a main effect of the *LRP-1* SNPs on BMI, regression analysis was conducting using the “PROC MIXED” command with genotypes modeled as a dominant effect, age and age^2^, sex, smoking (measured as current smoker or non-smoker), average total alcohol consumption/day, and center of data collection as predictors, and BMI (logarithmically transformed) as the outcome. Pedigree membership was treated as a random effect. To test for gene-gene interactions, the same model was run, and we additionally included *APOE* genotype as a predictor and an interaction term between *LRP-1* and *APOE* genotypes. To control for differences in group variances a Kenward Roger approximation to the degrees of freedom for the reference distribution was applied [16].

## Results

### Participant characteristics

Table 1 shows allele frequencies, *APOE* genotype frequencies, age and sex by LRP-1 genotype.

**Table 1:** N, mean age (standard deviation) and percentage of males and ApoE ε3 carriers, in the GOLDN study population by LRP-1 genotype group.

	Genotypes†			*P* ‡
	aa	aA	AA	
*C766T*				
N	16	246	771	
Gender, % male	31.25	45.93	48.9	0.29
Age, y	48.83 (14.84)	49.55 (16.12)	48.50 (16.24)	0.68
Current smokers, %	0	7.32	7.52	0.52
Alcohol intake, g/day	3.42 (2.69)	8.08 (29.53)	5.56 (16.54)	0.16
*I10701*				
N	110	428	493	
Gender, % male	50.00	48.83	46.45	0.69
Age, y	49.48 (17.69)	47.57 (15.94)	49.65 (16.02)	0.13
Current smokers, %	4.55	8.18	7.30	0.43
Alcohol intake, g/day	4.87 (9.09)	5.44 (18.41)	6.97 (23.50)	0.42
*I68477*				
N	91	456	483	
Gender, % male	48.24	47.37	47.25	0.96
Age, y	48.33 (15.88)	49.13 (16.59)	49.07 (15.51)	0.73
Current smokers, %	12.09	7.24	6.63	0.19
Alcohol intake, g/day	3.24 (8.16)	6.06 (21.25)	6.70 (21.05)	0.33

† a = minor allele; A = major allele.

‡ *P*-values were derived from tests of null hypothesis that no group is different, using a 1-way ANOVA for continuous traits or the χ^2^ test for categorical variables.

### Main effects of LRP-1 on BMI

An examination of BMI by genotype frequencies suggested dominant modes of inheritance for the effects of LRP-1 on BMI (Table 2). In mixed linear models that compared major allele carriers to those homozygous for the minor allele, while controlling for the covariates previously described, the rs1799986 and rs1800191 SNPs were not significantly associated with BMI (P>.05; Table 2). However, carriers of the major allele of rs715948 had BMIs, on average, 1.03 kg/m^2^ higher than those homozygous for the minor allele (P = .03). However, this unadjusted P-value did not remain significant after a Bonferroni correction for multiple testing was applied.

**Table 2:** BMI (kg/m^2^; standard deviation) distribution of the GOLDN study population by *LRP-1* genotype.

	Genotypes^a^			*P* b	*P_adj_* c
	aa	aA	AA		
C766T	30.41 (4.54)	29.03 (5.64)	28.05 (5.67)	0.28	.76
**I10701**	**27.46 (5.84)**	**28.21 (5.65)**	**28.63 (5.62)**	**0.03**	**.09**
I68477	29.15 (5.29)	28.24 (5.76)	28.24 (5.61)	0.24	.72

a a = minor allele; A = major allele.

b *P*-values were derived from mixed linear models with genotype frequency, age and age^2^, sex, smoking, total alcohol and center of data collection as predictors, and BMI (logarithmically transformed) as the outcome within a dominant model (AA/Aa vs. aa). BMI Data are presented that are back transformed for easy interpretation.

c P-value adjusted after a Bonferroni correction.

### Interactions between LRP-1 rs715948 and APOE genotypes on BMI

Frequencies of ε2 carriers and ε3/ε3 genotypes by *LRP-1* rs715948 locus genotypes are shown in Table 3. For those who carried the effect of the major allele at the *LRP-1 rs715948 locus*, no significant interaction was observed with ApoE genotype (Table 4).

**Table 3:** ApoE genotypes by LRP-1 *I10701* genotype.

	Genotypes†			*P*‡
	aa	aA	AA	
ε2 carriers; %	11.82	10.28	10.34	.89
ε4 carriers; %	27.27	25.93	25.35	.91
ε3/ε3 carriers; %	58.18	59.81	59.23	.95

**Table 4:** Parameter estimates from linear regression models looking at the effects of *LRP-1* I10701 SNP and *ApoE* isoforms on BMI in the GOLDN study population.

	Beta (β)	SE	*P* a
*ε2 carriers vs. ε3/ε3 ApoE genotype*			
LRP-1 I10701 genotype	−0.01	0.01	0.26
ApoE isoform	0.004	0.01	0.29
Interaction term	−0.02	0.02	0.93
*ε4 carriers vs. ε3/ε3 ApoE genotype*			
LRP-1 genotype	−0.02	0.01	0.01
ApoE isoform	0.03	0.01	0.52
**Interaction term**	**−0.02**	**0.02**	**0.28**

a *P*-values were derived from mixed linear models, specifying a Kenward Rogers correction on the estimator, with *LRP-1* I10701 SNP and *ApoE* genotype frequency, an interaction between ApoE and LRP-1 genotype frequency, age and age^2^, sex, smoking, total alcohol and center of data collection as predictors, and BMI (logarithmically transformed) as the outcome.

## Discussion

In a large, epidemiological cohort we examined three SNPs on the *LRP-1* gene for associations with BMI, and an interaction with ApoE genotype that may modify this association. We report that overall, the major allele of the rs715948 SNP was associated with BMIs 1.03 kg/m2 higher than those without the major allele, and that this relationship is not modified by differences in ApoE genotype.

Previously, *LRP-1* knockout mice have been associated with poorer TGRL clearance, although the direction of effect on BMI is not clear. Hoffman *et al* report *LRP-1* knockout mice to have a lower fat mass, which in addition to the observed elevated energy expenditure, was attributed reduced postprandial TGRL uptake [4]. However, although Liu *et al* report similar perturbations in postprandial TGRL clearance, they found *LRP-1* knockout mice to be associated with a 2-fold increase in fat mass compared to wild-type mice, coinciding with an ‘obese phenotype’ which included increased food intake, reduced energy expenditure and decreased leptin [5]. Their association of *LRP-1* knockout mice with increased fat mass was supported by work by Terrand *et al*, who found *LRP-1* knockout mice to show increased body fat arising from reduce lipolysis [6]. Although *LRP-1* has been shown to be upregulated in obese human adipose tissue [3], and increased concentrations of TGRL, such as chylomicron remnants, have been implicated in obesity in humans [7], this is the first study, to our knowledge, to report an association between *LRP-1* variants and BMI in humans. We report a significant association between SNP rs715948 in intron 2 of the *LRP-1* gene (*P* = .03). This SNP is in the cholesterol-lowering pathway, has previously been associated with cholesterol responses to statin therapy [17], but not to other lipid or obesity-related phenotypes in humans. Animal models suggest that impaired receptor-mediated TGRL chylomicron remnant clearance in conjunction with decreased energy expenditure and lipolysis may, in part, explain the 1.03 difference in BMI we observed between those homozygous for the minor allele and carriers of the major allele at the I10701 locus on the *LRP-1*.

We observed that the association between the LRP-1 rs715948 SNP was not significantly modified by carrying either the ε2 nor the ε4 allele at the *ApoE* locus. Therefore, the binding of the LRP-1 receptor to the isoform of ApoE on the chylomicron is not suggested as the mechanism by which LRP-1 variants affect BMI. A closer examination of postprandial TGRL would confirm such findings.

Our analysis was limited to white Americans of European descent and it is not clear if these results generalize to other ethnicities. In addition, replication is necessary as the finding of a main effect of *LRP-1* on BMI did not survive the correction for multiple testing. Despite these limitations we provide evidence that the *LRP-1* locus contributes to variations in BMI, and that these *LRP-1* variations are not mediated by variations at the *ApoE* locus in this relationship. This information is useful in starting to understand the biological causes to differences in BMI, and warrants replication in independent samples.
